# Evaluating the impact of environmental interventions across 2 countries: the International Bikeshare Impacts on Cycling and Collisions Study (IBICCS) Study protocol

**DOI:** 10.1186/1471-2458-14-1103

**Published:** 2014-10-25

**Authors:** Daniel Fuller, Lise Gauvin, Anne-Sophie Dubé, Meghan Winters, Kay Teschke, Elizabeth T Russo, Andi Camden, Carol Mee, Steven Marc Friedman

**Affiliations:** Department of Community Health and Epidemiology, University of Saskatchewan, Kragujevac, Saskatchewan Canada; Research Centre CHUM, Université de Montréal, Montréal, Canada; Département de médecine sociale et préventive, École de santé publique, Université de Montréal, Montréal, Canada; Direction de santé publique de l’Agence de la santé et des services sociaux de Montréal, Montréal, Canada; Faculty of Health Sciences, Simon Fraser University, Burnaby, Canada; School of Population and Public Health, University of British Columbia, Vancouver, Canada; Boston Public Health Commission, Boston, Massachusetts USA; Healthy Public Policy, Toronto Public Health, Toronto, Canada; Emergency Medicine, Toronto University Health Network, Toronto, Canada; Faculty of Medicine, University of Toronto, Toronto, Canada

**Keywords:** Natural experiment, Bicycle share program, Population health, Protocol, Evaluation

## Abstract

**Background:**

Few international studies examine public bicycle share programs (PBSP) health impacts. We describe the protocol for the International Bikeshare Impacts on Cycling and Collisions Study (IBICCS).

**Methods:**

A quasi-experimental non-equivalent groups design was used. Intervention cities (Montreal, Toronto, Boston, New York and Vancouver) were matched to control cities (Chicago, Detroit, and Philadelphia) on total population, population density, cycling rates, and average yearly temperature. The study used three repeated, cross-sectional surveys in intervention and control cities in Fall 2012 (baseline), 2013 (year 1), and 2014 (year 2). A non-probabilistic online panel survey with a sampling frame of individuals residing in and around areas where PBSP are/would be implemented was used. A total of 12,000 respondents will be sampled. In each of the 8 cities 1000 respondents will be sampled with an additional 4000 respondents sampled based on the total population of the city. Survey questions include measures of self-rated health, and self-reported height and weight, knowledge and experience using PBSP, physical activity, bicycle helmet use and history of collisions and injuries while cycling, socio-demographic questions, and home/workplace locations. Respondents could complete questionnaires in English, French, and Spanish. Two weights will be applied to the data: inverse probability of selection and post-stratification on age and sex.

A triple difference analysis will be used. This approach includes in the models, time, exposure, and treatment group, and interaction terms between these variables to estimate changes across time, between exposure groups and between cities.

**Discussion:**

There are scientific and practical challenges in evaluating PBSP. Methodological challenges included: appropriate sample recruitment, exchangeability of treatment and control groups, controlling unmeasured confounding, and specifying exposure. Practical challenges arise in the evaluation of environmental interventions such as a PBSP: one of the companies involved filed for bankruptcy, a Hurricane devastated New York City, and one PBSP was not implemented. Overall, this protocol provides methodological and practical guidance for researchers wanting to study PBSP impacts on health.

**Electronic supplementary material:**

The online version of this article (doi:10.1186/1471-2458-14-1103) contains supplementary material, which is available to authorized users.

## Background

Public bicycle share programs (PBSP) are a promising environmental intervention that deploy bicycles at docking stations throughout a city or territory [1]. Increasingly popular around the world, PBSP have grown from five programs in Europe in 2000 to an estimated 600,000 bicycles across 636 programs in 49 countries in 2013 [2]. Despite rapid expansion, a recent review suggests that the impacts of these programs on health are poorly understood [3]. Preliminary studies of PBSP show that implementation is associated with increased levels of physical activity [4] without increased risk of collisions between cyclists and motor vehicles [2, 5]. Safety features may differ between PBSP and personal bike use. For example PBSP bikes have daytime running lights, wide tires, and gearing that does not facilitate high speed travel [3, 6]. However, PBSP users are less likely to wear helmets [4, 7]. There are a number of scientific and practical challenges that have limited rigorous evaluations of PBSP impacts on health.

A first challenge is that studies examining PBSP programs are often case studies conducted in one city. There have been few attempts to generalize results by studying multiple cities simultaneously. Second, many cycling studies do not meet scientific criteria for internal validity, [8, 9] because PBSP interventions are generally implemented outside of the research sector. It is difficult for researchers to undertake the necessary steps (i.e., study design, ethics, funding) for evaluation prior to implementation [10, 11]. As a result, the overall validity, and particularly the internal validity of these studies are often wanting. For example, studies may not include pre-intervention measures or control groups. Finally, few publications provide study protocols describing the study design in detail making replication by independent research groups challenging.

Given the need for more research examining the potential health impacts, both positive and negative, of PBSP and the dearth of published protocols, we present the protocol for the International Bikeshare Impacts on Cycling and Collisions Study (IBICCS). The IBICCS study aims to examine the impact of PBSP on population-levels of cycling and risk of collisions between cyclists and motor vehicles in North America. We also discuss the methodological challenges for future studies examining PBSP health impacts.

## Methods

### Design

A quasi-experimental non-equivalent groups design was used [12]. This design is similar to a pretest-posttest randomized experiment but lacks randomization. Rather, intact groups (in this case, populations living in different cities) are used as treatment and control groups. The primary advantages of the non-equivalent groups design over past research are the pre-post measurement and inclusion of comparison groups [13, 14]. The pre-post measurement controls for potential reverse causality and factors that do not change within cities over time. Comparison groups allow for a plausible contrast to be made between cities. In randomized designs, it is assumed that treatment and control groups are exchangeable on all measured and unmeasured factors, on average, except the intervention. Because we used propensity score matching (discussed below), we assumed that cities were exchangeable, on average, on measured factors included in the propensity score analysis. Ethical approval was granted by the "*Centre de Recherche du Centre Hospitalier de l’Université de Montréal."*

### Intervention and control cities

Intervention cities were Montreal, Toronto, Boston, New York, and Vancouver. Intervention cities either had an existing PBSP (Montreal, Toronto & Boston) or a planned implementation in Spring 2012 (New York) or 2013 (Vancouver). To identify control cities, we used propensity score matching with replacement based on total population, population density, cycling rates, and average yearly temperature from the 60 largest cities in the United States and Canada based on data from the 2010 US census, 2008 American Community Survey, 2006 Canadian Census, Environment Canada, and US National Weather Service. One to one propensity score matching identified intervention and control cities similar on observable characteristics [15]. Matching with replacement ensured the best match, which is of critical importance for causal inference given the small number and variability between cities. Control cities were Chicago (comparison for New York, Montreal, Toronto), Detroit (for Boston), and Philadelphia (for Vancouver). STATA’s "psmatch2" command was used to conduct the matching analysis [16].

### Sampling plan

The study used three repeated, cross-sectional surveys in intervention and control cities in Fall 2012 (baseline), 2013 (year 1), and 2014 (year 2). We conducted repeated, cross sectional surveys rather than a longitudinal design (i.e., repeated measures of individuals across time) because the interest is in outcomes at the population level and to estimate population prevalence of PBSP use across time. Surveys were intended to be administered starting on November 1 of each year with 3–4 weeks anticipated for data collection. Prior to the baseline survey Hurricane Sandy struck New York between October 26 and October 29, 2012. Data collection was delayed for the baseline survey until November 12, 2012. There is discrepancy between data collection periods between the first (November 12 to December 12, 2012) and second (November 1 to November 22, 2013) surveys.

The sampling frame for the study was individuals aged 18+ years residing in and around areas where PBSP stations are (or would) be implemented during the study period. Forward Sortation Areas (FSA, Canada) and ZIP codes (USA) defined the sampling areas in each city.

A polling firm drew the sample from a non-probabilistic online panel survey operated by a private company. Online panel surveys are constructed for the purpose of participating in future surveys, typically for a monetary incentive. Anyone may choose to become a panel member and must provide verifiable information about their personal characteristics (e.g., age, sex, place of residence, education and income category) [17]. Respondents provided informed consent online by clicking a required check box. Respondents were offered a small financial incentive ($1-2 or equivalent points at retailers) for survey completion.

A proportional sampling approach was used in each city. The sampling plan called for a total of 12,000 respondents to be sampled. In each of the 8 cities 1000 respondents were sampled with an additional 4000 respondents to be sample based on the total population of the city. Table 1 shows the total population, sampling frame population (N), and the targeted sample size (n) for each city.

**Table 1:** **Characteristics of intervention and control cities and targeted sampling plan for the International Bikeshare Impacts on Cycling and Collisions Study (IBICCS), 2012-2014**

	City							
	Intervention					Control		
	Montreal ^+^	Toronto ^+^	Vancouver ^+^	New York ^~^	Boston* ^~^	Chicago ^~^	Detroit ^~^	Philadelphia ^~^
Population 2012	3,917,900	5,841,100	2,426,200	8,336,697	802,065	2,697,843	701,475	1,547,607
Sample frame population (N)	986,565	1,577,685	773,300	2,754,792	277,184	1,275,196	403,621	606,330
Targeted sample (n)	1458	1723	1168	2136	1142	1750	1198	1424
Sampling fraction ((n/N)*100))	0.15	0.11	0.15	0.08	0.41	0.14	0.30	0.23
Average yearly temperature	11.1	12.5	13.7	12.5	10.8	9.3	9.7	12.8
Cycling as usual mode of transportation to work %	1.6	1.0	1.7	0.8	1.4	1.3	0.3	1.8
Population density (square Km)	854	866	736	10,194	4,697	4,923	2,647	4,337
FSA\ZIP number	56	70	35	85	42	65	18	19

Notes.

*Includes the populations of Boston, Cambridge and Brookline, Massachusetts.

^+^Statistics Canada Census Metropolitan areas.

( http://www.statcan.gc.ca/tables-tableaux/sum-som/l01/cst01/demo05a-eng.htm).

^~^ Incorporated Places and Minor Civil Divisions Datasets.

( http://www.census.gov/popest/data/cities/totals/2012/SUB-EST2012.html).

### Questionnaire

The questionnaire included 5 sections (see Additional file 1); health status, including self-rated health, and self-reported height and weight; knowledge and experience using PBSP; physical activity, using the International Physical Activity [18] and the US Behavioral Risk Factor Surveillance System questionnaires [19]; bicycle helmet use and history of collisions and injuries while cycling; and standard socio-demographic questions from the Statistics Canada and US Census questionnaires; and information about home and workplace locations. Respondents could complete questionnaires in English, French, and Spanish. Questionnaires were developed by the research team in English and French, and translated to Spanish.

### Quantifying exposure

Exposure to the PBSP for intervention and control cities will be quantified two ways. In control cities we will generate a hypothetical bicycle share program. This will be done based on the criteria of the US Department of Transportation (USDOT), namely, deployed in areas of high population and job density and public transportation connectivity, no more than 500 meters apart, and in locations that are clearly visible [20]. The cities are matched on population density and other characteristics. Within cities PBSP are implemented where population and retail density, and cycling rates are higher. Creating hypothetical station locations in control cities will allow a more precise examination of characteristics within cities where PBSP are deployed or would be deployed.

Using actual (intervention cities) or hypothetical (control cities) station locations, exposure will be defined based on the number of stations within a 500 meter road network buffer of a respondent’s home, and workplace. An individual’s exposure will be defined in 4 categories: i) fully exposed - at least one station in both their home and work buffers; ii) work exposed - at least one station within their work buffer but no stations within their home buffer; iii) home exposed - at least one station within their home buffer but no stations within their work buffer; and iv) non exposed - no stations at home and work.

### Weighting

Two weights will be applied to the data: inverse probability of selection and post-stratification on age and sex. Inverse probability of selection weights correct for unequal sampling fractions as not everyone is equally likely to be sampled in each city and area [21]. Post-stratification weights on age and sex use valid surveys (census) to adjust the sample characteristics to represent the best estimate of the population [22]. Given that the online panel survey is non-probabilistic using these weights is recommended to reduce sampling bias [17].

### Analysis plan

The proposed data analysis is drawn from techniques which approximate randomized controlled trials in quasi-experiments [23]. A triple difference analysis will be used [24]. This approach includes in the models, time (baseline, year 1, and year 2), exposure (4 categories), and treatment (intervention and control), and interaction terms between these variables to estimate changes across time, between exposure groups and between cities [14, 25]. We will estimate PBSP impacts on cycling, physical activity, and collisions, across time, between exposed and unexposed, and between cities. We will be able to examine the overall impact of PBSP on health and the specific impact in each city.

## Discussion

### Treatment/control groups

A particular challenge for this study is the rapid expansion of PBSP in North America. In June 2013 Chicago, a planned control city, launched the PBSP Divvy ( http://divvybikes.com/) with 750 bikes at 75 stations. In Fall 2014, Philadelphia, a planned control city, will launch a PBSP ( http://www.phila.gov/bikeshare/Pages/default.aspx). Vancouver, which had a planned implementation in 2013, was delayed and may not launch during the study period. Despite our efforts to identify intervention and control groups similar on observable characteristics, new implementations and delays of programs mean that by the time data is collected cities may have switched from intervention to control and our exchangeability assumptions will no longer be plausible.

### Between country differences

The international nature of the study introduces challenges. In particular, differences between administrative boundaries used for sampling and defining exposure (FSA vs. ZIP Code vs. Census Tract) may lead to potential bias [26]. The average population of a FSA was 20,463, compared with 30,000 for ZIP codes. Canadian census tracts include 2,500 - 8,000 persons, whereas US census tracts include 1,200 - 8,000 people, and an optimum size of 4,000 people. As a result of these geographic differences, modifiable area unit problems are of particular concern [27, 28].

### Expanded data collection areas

**Figure 1:**
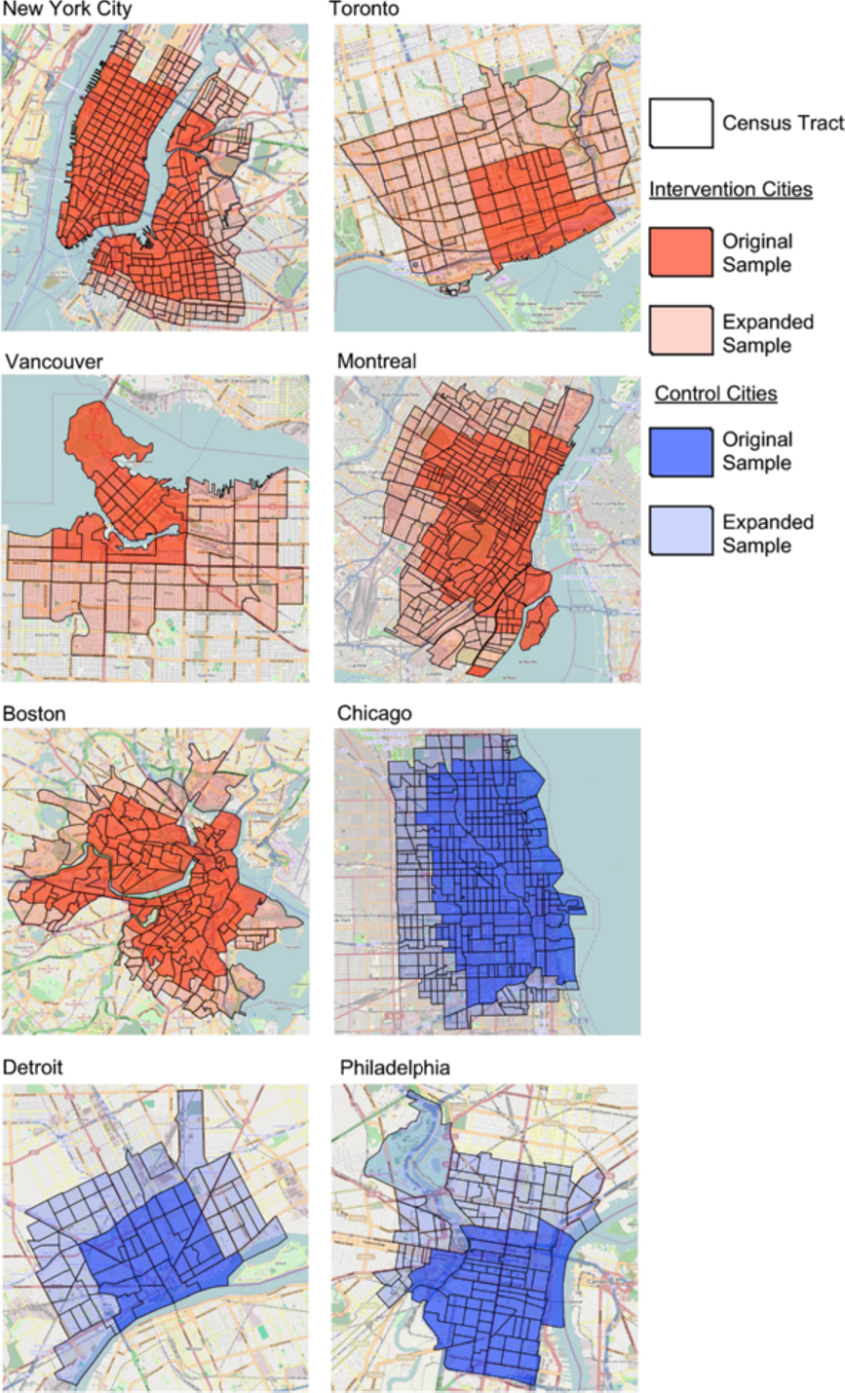
**Sampled areas for intervention and control cities in the International Bikeshare Impacts on Cycling and Collisions Study (IBICCS), 2012–2014.** Note. Basemap is from Open Street Maps (http://www.openstreetmap.org/).

During the pre-implementation data collection period, we had planned to recruit from a specific set of FSA and ZIP codes. These areas did not generate a sufficiently large sample. After up to 2 reminder emails, we expanded data collection areas on November 30, 2012 to include additional geographic areas. Figure 1 shows the geographic areas added.

### Bankruptcy BIXI Montreal

On January 21 2014, the Montreal-based PBSP filed for bankruptcy protection [29]. This announcement has implications for the study as the Montreal company is an operator or supplier for many PBSP in North America. Although media coverage in New York [30], Vancouver [31], and Chicago [32] suggest that bankruptcy should not affect operations, its ramifications on implementation, impact, and public perception remain an unknown.

### Data sharing

This project will involve data from several thousand respondents including information on individual health, socio-economic, and environmental exposure data. The project will involve sharing de-identified data with partners from each city and allow for comparisons between cities and local case studies to inform policies around PBSP.

## Conclusion

By introducing the study protocol we hope to stimulate other research groups to replicate and extend the design to ensure comparability between studies, and more valid and generalizable conclusions about the health impacts of implementing PBSP.

## Electronic supplementary material

Additional file 1: **Appendix.** (PDF 851 KB)
