# Supplementary material for: Evaluating the impact of environmental interventions across 2 countries: the International Bikeshare Impacts on Cycling and Collisions Study (IBICCS) Study protocol
Source: BMC Public Health. 2014 Oct 25;14:1103. doi: 10.1186/1471-2458-14-1103 (PMC4232659; doi:10.1186/1471-2458-14-1103)
Supplement: Supplementary file 1 — Additional file 1: Appendix. (PDF 851 KB) [file 12889_2014_7221_MOESM1_ESM.pdf]

4 LANG

LANGUE D'ENTREVUE

N =		0	100%
Français	FR	0	0%
Anglais	EN	0	0%
Espagnol	ES	0	0%

5 VILLE

In which city do you live?

N =		0	100%
New-York	01	0	0%
Chicago	02	0	0%
Toronto	03	0	0%
Vancouver	04	0	0%
Detroit	05	0	0%
Montreal	06	0	0%
Boston	07	0	0%
Philadelphie	08	0	0%
Other (specify:)	96O	0	0%

12 Q1

For statistical purposes, can you please provide your postal code or zip code. Please note that this information does not allow for identification of you as an individual but allows us to better understand what part of the city you live in and what kind of services you have access to. Please do not enter any spaces.

A9A9A9

23 Q46B9

What is the name of the neighborhood where you live?

N =		0	100%
Other (Specify:)	96O	0	0%

24 Q42

How old are you?

25 Q54

Are you…?

N =		0	100%
Female	1	0	0%
Male	2	0	0%

Refuse	9	0	0%

26			Q43

What language do you most often speak at home?

N =		0	100%
English	01	0	0%
French	02	0	0%
Spanish	03	0	0%
Other, specify	96O	0	0%
Refuse	99	0	0%

36			Q2

In this first series of questions, we ask about your current health status. Please indicate the answer that best describes your current state of health. In comparison to other persons your age, would you say that your health in general is...

N =		0	100%
Excellent	1	0	0%
Very good	2	0	0%
Good	3	0	0%
Fair	4	0	0%
Poor	5	0	0%
Do not know/Does not apply	9	0	0%

37			Q3B

Below is a list of symptoms and conditions. For each one, please indicate whether or not a medical doctor, nurse or other professional had ever told you that you suffer from this symptom or condition.

Hypertension

N =		0	100%
Yes	1	0	0%
No	2	0	0%
Do not know/Does not apply	3	0	0%

38			Q3C

Below is a list of symptoms and conditions. For each one, please indicate whether or not a medical doctor, nurse or other professional had ever told you that you suffer from this symptom or condition.

High cholesterol

N =		0	100%
Yes	1	0	0%
No	2	0	0%

Do not know/Does not apply	3	0	0%

39 Q3D

Below is a list of symptoms and conditions. For each one, please indicate whether or not a medical doctor, nurse or other professional had ever told you that you suffer from this symptom or condition.

Diabetes

N =		0	100%
Yes	1	0	0%
No	2	0	0%

40 Q3E

Below is a list of symptoms and conditions. For each one, please indicate whether or not a medical doctor, nurse or other professional had ever told you that you suffer from this symptom or condition.

Cardiac problems (angina, heart attack/myocardial infarction, by-pass)

N =		0	100%
Yes	1	0	0%
No	2	0	0%
Do not know/Does not apply	3	0	0%

41 Q3F

Below is a list of symptoms and conditions. For each one, please indicate whether or not a medical doctor, nurse or other professional had ever told you that you suffer from this symptom or condition.

Arthritis/ or rheumatism

N =		0	100%
Yes	1	0	0%
No	2	0	0%
Do not know/Does not apply	3	0	0%

42 Q3G

Below is a list of symptoms and conditions. For each one, please indicate whether or not a medical doctor, nurse or other professional had ever told you that you suffer from this symptom or condition.

Respiratory illness (asthma, COPD-Chronic Obstructive Pulmonary Disease)

N =		0	100%
Yes	1	0	0%
No	2	0	0%
Do not know/Does not apply	3	0	0%

43			Q3H

Below is a list of symptoms and conditions. For each one, please indicate whether or not a medical doctor, nurse or other professional had ever told you that you suffer from this symptom or condition.

Depression

N =		0	100%
Yes	1	0	0%
No	2	0	0%
Do not know/Does not apply	3	0	0%

44			Q3I

Below is a list of symptoms and conditions. For each one, please indicate whether or not a medical doctor, nurse or other professional had ever told you that you suffer from this symptom or condition.

Back or neck problems

N =		0	100%
Yes	1	0	0%
No	2	0	0%
Do not know/Does not apply	3	0	0%

45			Q4

In this next series of questions, we ask about your knowledge of a specific service which is being implemented in many North American cities. Public bicycle share programs are public bicycle systems, often operated through the City, that give people access to bikes for temporary travel around a city. The bikes are often stationed at docking stations throughout the busiest downtown areas. Here are the names of some of these programs in North America and in Europe: Velib in Paris France, Boris Bikes in London England, BIXI bikes in Montreal and Toronto Canada, Capital Bike Share in Washington DC, Nice Ride in Minneapolis Minnesota, Hubway in Boston Massachusetts, Bike Share in NYC EcoBici in Mexico City

Have you heard about one or more of these bike share programs?

N =		0	100%
Yes	1	0	0%
No	2	0	0%
Do not know/Does not apply	9	0	0%

46			Q5

Have you ever used one of these public share bicycles in the city in which you live?

N =		0	100%

Yes	1	0	0%
No	2	0	0%
Do not know/Does not apply	9	0	0%

47	Q6

Please indicate how many times per day, per week, per month or per year have you used these public rented bicycles? Please do not fill out or check more than one box.

N =		0	100%
Per day	1DI	0	0%

48	Q6A

...Per day

Mean =	0.000	
Standard deviation =	0.000	
N =	0	100%

49	Q6B

...Per week

Mean =	0.000	
Standard deviation =	0.000	
N =	0	100%

50	Q6C

...Per month

Mean =	0.000	
Standard deviation =	0.000	
N =	0	100%

51	Q6D

...Per year

Mean =	0.000	
Standard deviation =	0.000	
N =	0	100%

56	Q7

For how many minutes or hours did you use these bikes on average per trip? Please do not fill out or check more than one box.

N =		0	100%
Minutes	1DI	0	0%

57 Q7A

...in minutes?

Mean =	0.000	
Standard deviation =	0.000	
N =	0	100%

58 Q7B

...in hours?

Mean =	0.000	
Standard deviation =	0.000	
N =	0	100%

59 Q7C

N =		0	100%
Don’t know/Not sure	3	0	0%
Refused	9	0	0%

63 Q8

When you used these bicycles what type of trip did it normally involve? (Check all that apply)

Getting to work or school	01	0	0%
For leisure or fun	02	0	0%
For exercise	03	0	0%
To do shopping or run other errands	04	0	0%
To meet with family or friends	05	0	0%
Trips for work	06	0	0%
Other, please specify	96O	0	0%
Do not know/Does not apply	99X	0	0%

64 Q9

What type of transportation would you have used to make these trips if these public share bicycles were not available? (Check all that apply)

Personal bike	01	0	0%
Walk	02	0	0%
Public transit	03	0	0%
Taxi	04	0	0%
Motor vehicle (own, rented, borrowed, car pool)	05	0	0%
Other, please specify	96O	0	0%
Do not know/Does not apply	99X	0	0%

65 Q10

Which of the following modes of transportation do you integrate into your travel when you use public share bicycles? (Check all that apply)

N =		0	100%
Public transit	01	0	0%
Motor vehicule	02	0	0%
Taxi	03	0	0%
Other, please specify	96O	0	0%
None of the above	98X	0	0%
Do not know/Does not apply	99X	0	0%

66 Q11

If public bicycles share were located within a 10-minute walk from your home, workplace or school, how likely would you be to rent a public share bicycle?

Very likely	1	0	0%
Somewhat likely	2	0	0%
Somewhat unlikely	3	0	0%
Totally unlikely	4	0	0%
Don't know	5	0	0%
I already use public bike share	9	0	0%

67 Q12

Which of the following reasons best represent why you are *q11*? (Check all that apply)

Prefer to ride your own bicycle	01	0	0%
Cycling is unsafe	02	0	0%
The cost of renting a public bicycle is too expensive	03	0	0%
Public bicycle does not offer helmet rentals	04	0	0%
A credit card is required to rent a public bicycle	05	0	0%
A security deposit (on a credit card) is	06	0	0%

required to rent a public bicycle			
My destination is too far	07	0	0%
I am not interested in cycling	08	0	0%
Public bicycle time restrictions	09	0	0%
There are not enough public bicycles at each docking station	10	0	0%
Other types of transpsortation are more convenient	11	0	0%
Other, please specify	96O	0	0%
None of the above	98X	0	0%
Don't know	99X	0	0%

68 **Q13**

Overall, which mode of transportation do you use most often to get around?

Personal bike	01	0	0%
Public bicycle share	02	0	0%
Walk	03	0	0%
Bus, subway, train	04	0	0%
Taxi	05	0	0%
Motor vehicle (own, rented, borrowed, car pool)	06	0	0%
Not applicable	97	0	0%
Other, please specify	96O	0	0%
Do not know	99	0	0%

69 **Q14**

The next few questions are about physical activities other than your regular job duties. During the past month, other than your regular job, did you participate in any physical activities or exercises such as running, calisthenics, golf, gardening, or walking for exercise? ***ny***

N =		0	100%
Yes	1	0	0%
No	2	0	0%
Do not know/Does not apply	8	0	0%
Refused	9	0	0%

70 **Q15**

What type of physical activity or exercise did you spend the most time doing during the past month? ***ny***

Active Gaming Devices (Wii Fit, Dance Dance revolution)	01	0	0%

Aerobics video or class	02	0	0%
Backpacking	03	0	0%
Badminton	04	0	0%
Basketball	05	0	0%
Bicycling machine exercise	06	0	0%
Bicycling	07	0	0%
Boating (Canoeing, rowing, kayaking,sailing for pleasure or camping)	08	0	0%
Bowling	09	0	0%
Boxing	10	0	0%
Calisthenics	11	0	0%
Canoeing/rowing in competition	12	0	0%
Carpentry	13	0	0%
Dancing-ballet, ballroom, Latin, hip hop, etc	14	0	0%
Elliptical/EFX machine exercise	15	0	0%
Fishing from river bank or boat	16	0	0%
Frisbee	17	0	0%
Gardening (spading, weeding, digging, filling)	18	0	0%
Golf (with motorized cart)	19	0	0%
Golf (without motorized cart)	20	0	0%
Handball	21	0	0%
Hiking - cross-country	22	0	0%
Hockey	23	0	0%
Horseback riding	24	0	0%
Hunting large game - deer, elk	25	0	0%
Hunting small game - quail	26	0	0%
Inline Skating	27	0	0%
Jogging	28	0	0%
Lacrosse	29	0	0%
Mountain climbing	30	0	0%
Mowing lawn	31	0	0%
Paddleball	32	0	0%
Painting/papering house	33	0	0%
Pilates	34	0	0%
Racquetball	35	0	0%
Raking lawn	36	0	0%
Running	37	0	0%
Rock Climbing	38	0	0%
Rope skipping	39	0	0%
Rowing machine exercise	40	0	0%
Rugby	41	0	0%
Scuba diving	42	0	0%
Skateboarding	43	0	0%
Skating - ice or roller	44	0	0%

Sledding, tobogganing	45	0	0%
Snorkeling	46	0	0%
Snow blowing	47	0	0%
Snow shoveling by hand	48	0	0%
Snow skiing	49	0	0%
Snowshoeing	50	0	0%
Soccer	51	0	0%
Softball/Baseball	52	0	0%
Squash	53	0	0%
Stair climbing/Stair master	54	0	0%
Stream fishing in waders	55	0	0%
Surfing	56	0	0%
Swimming	57	0	0%
Swimming in laps	58	0	0%
Table tennis	59	0	0%
Tai Chi	60	0	0%
Tennis	61	0	0%
Touch football	62	0	0%
Volleyball	63	0	0%
Walking	64	0	0%
Waterskiing	65	0	0%
Weight lifting	66	0	0%
Wrestling	67	0	0%
Yoga	68	0	0%
Other (Specify:)	96DO	0	0%
Do not know/Does not apply	98	0	0%
Refused	99	0	0%

71 Q16

How many times per week or per month did you take part in this activity during the past month? Please do not fill out or check more than one box. ***ny***

N =		0	100%
Times per week	1DI	0	0%

72 Q16A

per week

Mean =	0.000	
Standard deviation =	0.000	
N =	0	100%

73 Q16B

per month

Mean =	0.000
Standard deviation =	0.000

N =		0	100%

74 Q16C

N =		0	100%
Do not know/Does not apply	8	0	0%
Refused	9	0	0%

75 TQ16A

Please do not fill out or check more than one box. Click on "Next" to revise your answer.

N =		0	100%
	1DI	0	0%

76 TQ16B

Please do not fill out or check more than one box. Click on "Next" to revise your answer.

N =		0	100%
	1DI	0	0%

78 Q17

And when you took part in this activity, for how many minutes or hours did you usually keep at it? ***ny***

N =		0	100%
Minutes	1DI	0	0%

79 Q17A

Hours

Mean =		0.000	
Standard deviation =		0.000	
N =		0	100%

80 Q17B

Minutes

Mean =		0.000	
Standard deviation =		0.000	
N =		0	100%
Do not know/Does not apply	98	0	0%
Refused	99	0	0%

83 Q18

What other type of physical activity gave you the next most exercise during the past month? ***ny***

Active Gaming Devices (Wii Fit, Dance Dance revolution)	01	0	0%
Aerobics video or class	02	0	0%
Backpacking	03	0	0%
Badminton	04	0	0%
Basketball	05	0	0%
Bicycling machine exercise	06	0	0%
Bicycling	07	0	0%
Boating (Canoeing, rowing, kayaking,sailing for pleasure or camping)	08	0	0%
Bowling	09	0	0%
Boxing	10	0	0%
Calisthenics	11	0	0%
Canoeing/rowing in competition	12	0	0%
Carpentry?	13	0	0%
Dancing-ballet, ballroom, Latin, hip hop, etc	14	0	0%
Elliptical/EFX machine exercise	15	0	0%
Fishing from river bank or boat?	16	0	0%
Frisbee?	17	0	0%
Gardening (spading, weeding, digging, filling)	18	0	0%
Golf (with motorized cart)	19	0	0%
Golf (without motorized cart)	20	0	0%
Handball	21	0	0%
Hiking - cross-country	22	0	0%
Hockey	23	0	0%
Horseback riding	24	0	0%
Hunting large game - deer, elk	25	0	0%
Hunting small game - quail	26	0	0%
Inline Skating	27	0	0%
Jogging	28	0	0%
Lacrosse	29	0	0%
Mountain climbing	30	0	0%
Mowing lawn	31	0	0%
Paddleball	32	0	0%
Painting/papering house	33	0	0%
Pilates	34	0	0%
Racquetball	35	0	0%
Raking lawn	36	0	0%

Running	37	0	0%
Rock Climbing	38	0	0%
Rope skipping	39	0	0%
Rowing machine exercise	40	0	0%
Rugby	41	0	0%
Scuba diving	42	0	0%
Skateboarding	43	0	0%
Skating - ice or roller	44	0	0%
Sledding, tobogganing	45	0	0%
Snorkeling	46	0	0%
Snow blowing	47	0	0%
Snow shoveling by hand	48	0	0%
Snow skiing	49	0	0%
Snowshoeing	50	0	0%
Soccer	51	0	0%
Softball/Baseball	52	0	0%
Squash	53	0	0%
Stair climbing/Stair master	54	0	0%
Stream fishing in waders	55	0	0%
Surfing	56	0	0%
Swimming	57	0	0%
Swimming in laps	58	0	0%
Table tennis	59	0	0%
Tai Chi	60	0	0%
Tennis	61	0	0%
Touch football	62	0	0%
Volleyball	63	0	0%
Walking	64	0	0%
Waterskiing	65	0	0%
Weight lifting	66	0	0%
Wrestling	67	0	0%
Yoga	68	0	0%
Other (Specify:)	96DO	0	0%
No other activity	97	0	0%
Do not know/Does not apply	98	0	0%
Refused	99	0	0%

84 **Q19**

How many times per week or per month did you take part in this activity during the past month? Please do not fill out or check more than one box. ***ny***

N =		0	100%
per week	1DI	0	0%

85 **Q19A**

Per week

Mean =	0.000

Standard deviation =		0.000	
N =		0	100%

86			Q19B
**Per month**			
Mean =		0.000	
Standard deviation =		0.000	
N =		0	100%

87			Q19C
N =		0	100%
Do not know/Does not apply	8	0	0%
Refused	9	0	0%

91			Q20
**And when you took part in this activity, for how many minutes or hours did you usually keep at it?** ***ny***			
N =		0	100%
Minutes	1DI	0	0%

92			Q20A
**Hours**			
Mean =		0.000	
Standard deviation =		0.000	
N =		0	100%

93			Q20B
**Minutes**			
Mean =		0.000	
Standard deviation =		0.000	
N =		0	100%

94			Q20C
Do not know/Does not apply	8	0	0%
Refused	9	0	0%

98			Q21

Now, think about how you traveled from place to place, including to places like work, stores, movies and so on. During the past month, on how many days, on average, did you travel in a motor vehicle like a car, car pool or car share use? ***ny***

Days per week

Mean =		0.000	
Standard deviation =		0.000	
N =		0	100%
Do not know/Does not apply	98	0	0%
Refused	99	0	0%

99 Q22A

How much time did you usually spend on one of those days traveling in a car, car pool or car share use? Please do not fill out or check more than one box. ***ny***

N =		0	100%
Minutes	1DI	0	0%

100 Q22B

Hours per day

Mean =	0.000	
Standard deviation =	0.000	
N =	0	100%

101 Q22C

Minutes per day

Mean =	0.000	
Standard deviation =	0.000	
N =	0	100%

102 Q22D

Hours per week

Mean =	0.000	
Standard deviation =	0.000	
N =	0	100%

103 Q22E

Minutes per week

Mean =	0.000	
Standard deviation =	0.000	
N =	0	100%

104 Q22F

N =	0	100%

Do not know/Does not apply	7	0	0%
Refused	9	0	0%

108 Q23

During the past month, on how many days, on average, did you travel in public transit like a train, bus, subway or tram? *ny*

Days per week

Mean =		0.000	
Standard deviation =		0.000	
N =		0	100%
Do not know/Does not apply	8	0	0%
Refused	9	0	0%

109 Q24

How much time did you usually spend on one of those days traveling in public transit like a train, bus, subway or tram? Please do not fill out or check more than one box. *ny*

N =		0	100%
Hours per day	1DI	0	0%

110 Q24B

Hours per day

Mean =	0.000	
Standard deviation =	0.000	
N =	0	100%

111 Q24C

Minutes per day

Mean =	0.000	
Standard deviation =	0.000	
N =	0	100%

112 Q24D

Hours per week

Mean =	0.000	
Standard deviation =	0.000	
N =	0	100%

113 Q24E

Minutes per week

Mean =		0.000	
Standard deviation =		0.000	
N =		0	100%

114			Q24F
N =		0	100%
Do not know/Does not apply	7	0	0%
Refused	9	0	0%

118			Q25

Now think only about the walking you did to travel to and from work, to do errands or to go from place to place. Only include walking that you did for at least 10 minutes at a time. During the past month, on how many days, on average, per week did you walk to go from place to place? Days per week. ***ny***

Mean =		0.000	
Standard deviation =		0.000	
N =		0	100%
Do not know/Does not apply	8	0	0%
Refused	9	0	0%

119			Q26

How much time did you usually spend on one of those days walking from place to place? Please do not fill out or check more than one box. ***ny***

N =		0	100%
Hours per day	1DI	0	0%

120			Q26B

Hours per day

Mean =		0.000	
Standard deviation =		0.000	
N =		0	100%

121			Q26C

Minutes per day

Mean =		0.000	
Standard deviation =		0.000	
N =		0	100%

122			Q26D

Hours per week

Mean =		0.000	
Standard deviation =		0.000	

N =	0	100%

123 Q26E

Minutes per week

Mean =	0.000	
Standard deviation =	0.000	
N =	0	100%

124 Q26F

N =		0	100%
Do not know/Does not apply	7	0	0%
Refused	9	0	0%

128 Q27

Now think only about the bicycling you did to travel to and from work, to do errands, or to go from place to place. Only include bicycling that you did for at least 10 minutes at a time. During the past month, on how many days, on average, per week did you bicycle to go from place to place? Days per week. *ny*

Mean =		0.000	
Standard deviation =		0.000	
N =		0	100%
Do not know/Does not apply	98	0	0%
Refused	99	0	0%

129 Q28

How much time did you usually spend on one of those days to bicycle from place to place? Please do not fill out or check more than one box. *ny*

N =		0	100%
Hours per day	1DI	0	0%

130 Q28B

Hours per day

Mean =	0.000	
Standard deviation =	0.000	
N =	0	100%

131 Q28C

Minutes per day

Mean =	0.000	
Standard deviation =	0.000	
N =	0	100%

132			Q28D
**Hours per week**			
Mean =		0.000	
Standard deviation =		0.000	
N =		0	100%

133			Q28E
**Minutes per week**			
Mean =		0.000	
Standard deviation =		0.000	
N =		0	100%

134			Q28F
**How much time did you usually spend on one of those days to bicycle from place to place?**			
N =		0	100%
Do not know/Does not apply	7	0	0%
Refused	9	0	0%

138			Q29
**Overall, how safe do you think bicycling is in your city? Would you say it is…**			
N =		0	100%
Very safe	1	0	0%
Somewhat safe	2	0	0%
Neither safe nor unsafe (neutral)	3	0	0%
Somewhat dangerous	4	0	0%
Very dangerous	5	0	0%
Don't know	9	0	0%

139			Q30A
**There are certain risks associated with cycling. How do the following possible safety issues influence your decision to cycle?**			
The risk of injury from car-bike collisions			
N =		0	100%
Much less likely to cycle	1	0	0%
Less likely to cycle	2	0	0%
No influence on my decision	3	0	0%
More likely to cycle	4	0	0%
Much more likely to cycle	5	0	0%
Don't know	9	0	0%

140			Q30B

There are certain risks associated with cycling. How do the following possible safety issues influence your decision to cycle?

The risk of bicycle theft

N =		0	100%
Much less likely to cycle	1	0	0%
Less likely to cycle	2	0	0%
No influence on my decision	3	0	0%
More likely to cycle	4	0	0%
Much more likely to cycle	5	0	0%
Don’t know	9	0	0%

141 Q30C

There are certain risks associated with cycling. How do the following possible safety issues influence your decision to cycle?

The risk from motorists who don’t drive safely near bicycles

N =		0	100%
Much less likely to cycle	1	0	0%
Less likely to cycle	2	0	0%
No influence on my decision	3	0	0%
More likely to cycle	4	0	0%
Much more likely to cycle	5	0	0%
Don’t know	9	0	0%

142 Q30D

There are certain risks associated with cycling. How do the following possible safety issues influence your decision to cycle?

The risk from cyclists who don’t ride safely

N =		0	100%
Much less likely to cycle	1	0	0%
Less likely to cycle	2	0	0%
No influence on my decision	3	0	0%
More likely to cycle	4	0	0%
Much more likely to cycle	5	0	0%
Don’t know	9	0	0%

143 Q30E

There are certain risks associated with cycling. How do the following possible safety issues influence your decision to cycle?

The route has on-road bicycle lanes on major roads for the entire distance

N =		0	100%
Much less likely to cycle	1	0	0%
Less likely to cycle	2	0	0%
No influence on my decision	3	0	0%

More likely to cycle	4	0	0%
Much more likely to cycle	5	0	0%
Don’t know	9	0	0%

144 Q30F

There are certain risks associated with cycling. How do the following possible safety issues influence your decision to cycle?

The route has bike signage and pavement markings on streets

N =		0	100%
Much less likely to cycle	1	0	0%
Less likely to cycle	2	0	0%
No influence on my decision	3	0	0%
More likely to cycle	4	0	0%
Much more likely to cycle	5	0	0%
Don’t know	9	0	0%

145 Q30G

There are certain risks associated with cycling. How do the following possible safety issues influence your decision to cycle?

The route has bicycle paths separated from traffic for the entire distance

N =		0	100%
Much less likely to cycle	1	0	0%
Less likely to cycle	2	0	0%
No influence on my decision	3	0	0%
More likely to cycle	4	0	0%
Much more likely to cycle	5	0	0%
Don’t know	9	0	0%

146 Q30H

There are certain risks associated with cycling. How do the following possible safety issues influence your decision to cycle?

Vehicles drive faster than *m30h*

N =		0	100%
Much less likely to cycle	1	0	0%
Less likely to cycle	2	0	0%
No influence on my decision	3	0	0%
More likely to cycle	4	0	0%
Much more likely to cycle	5	0	0%
Don’t know	9	0	0%

147 Q31

In the past month, when using your own bike how often did you wear a helmet?

N =		0	100%

Always	1	0	0%
Often	2	0	0%
Seldom	3	0	0%
Never	4	0	0%
Do not know/Does not apply	9	0	0%

148 Q32

In the past month, when using public share bicycles, how often did you wear a helmet?

N =		0	100%
Always	1	0	0%
Often	2	0	0%
Seldom	3	0	0%
Never	4	0	0%
Do not know/Does not apply	9	0	0%

149 Q33

How many times did you fall off your bike in the past 3 months for any reason?

Mean =	0.000	
Standard deviation =	0.000	
N =	0	100%

150 Q34A

Have you been involved in a crash in the past 3 months in which you fell off your bike because of …

A car door opening in front of you

N =		0	100%
Yes	1	0	0%
No	2	0	0%
Do not know/Does not apply	9	0	0%

151 Q34B

Have you been involved in a crash in the past 3 months in which you fell off your bike because of …

A collision with a motor vehicle (including car, SUV, truck, bus, motorcycle)

N =		0	100%
Yes	1	0	0%
No	2	0	0%
Do not know/Does not apply	9	0	0%

152 Q34C

Have you been involved in a crash in the past 3 months in which you fell off your bike because of …

A collision with another cyclist

N =		0	100%
Yes	1	0	0%
No	2	0	0%
Do not know/Does not apply	9	0	0%

153 Q34D

Have you been involved in a crash in the past 3 months in which you fell off your bike because of …

A collision with a pedestrian

N =		0	100%
Yes	1	0	0%
No	2	0	0%
Do not know/Does not apply	9	0	0%

154 Q34E

Have you been involved in a crash in the past 3 months in which you fell off your bike because of …

An hazard on the road (tracks, pothole, curb)

N =		0	100%
Yes	1	0	0%
No	2	0	0%
Do not know/Does not apply	9	0	0%

155 Q34F

Have you been involved in a crash in the past 3 months in which you fell off your bike because of …

A maneuver to avoid a collision

N =		0	100%
Yes	1	0	0%
No	2	0	0%
Do not know/Does not apply	9	0	0%

156 Q34G

Have you been involved in a crash in the past 3 months in which you fell off your bike because of …

You were distracted

N =		0	100%
Yes	1	0	0%
No	2	0	0%
Do not know/Does not apply	9	0	0%

157			Q34H

Have you been involved in a crash in the past 3 months in which you fell off your bike because of …

Other type of crash

N =		0	100%
Yes	1	0	0%
No	2	0	0%
Do not know/Does not apply	9	0	0%

158			Q35

During your most recent collision in the past 3 months were you injured?

N =		0	100%
Yes	1	0	0%
No	2	0	0%
Do not know/Does not apply	9	0	0%

159			Q36

During your most recent collision in the past 3 months, did you visit an emergency room because you were hurt/injured?

N =		0	100%
Yes	1	0	0%
No	2	0	0%
Do not know/Does not apply	9	0	0%

160			Q37

During your most recent collision in the past 3 months, were you cycling on a cycling lane?

N =		0	100%
Yes	1	0	0%
No	2	0	0%
Do not know/Does not apply	9	0	0%

161			Q38

During your most recent collision in the past 3 month, were you cycling with your own bicycle, a public share bicycle, or a rented/borrowed bicycle?

N =		0	100%
Own	1	0	0%
Public share bicycle	2	0	0%
Rented/borrowed	3	0	0%
Do not know/Does not apply	9	0	0%

162 Q39

During your most recent collision in the past 3 months, were you wearing a helmet?

N =		0	100%
Yes	1	0	0%
No	2	0	0%
Do not know/Does not apply	9	0	0%

163 Q40

These final questions ask about your individual characteristics and will be used for comparison purposes only. All information will remain strictly anonymous.About how tall are you without shoes? Please indicate your height in feet and inches (imperial system) OR in metres and centimetres (metric system).

N =		0	100%
ft / inches	1DI	0	0%

164 Q40A

Ft

Mean =	0.000	
Standard deviation =	0.000	
N =	0	100%

165 Q40B

Inches

Mean =	0.000	
Standard deviation =	0.000	
N =	0	100%

166 Q40C

Meters

Mean =	0.000	
Standard deviation =	0.000	
N =	0	100%

167 Q40D

Centimeters

Mean =	0.000	
Standard deviation =	0.000	
N =	0	100%

168 Q40E

N =		0	100%
Do not know/Does not apply	3	0	0%
Refused	4	0	0%

172 Q41

About how much do you weigh without shoes? Please do not fill out or check more than one box.

N =		0	100%
Pounds	1DI	0	0%

173 Q41A

Pounds

Mean =	0.000	
Standard deviation =	0.000	
N =	0	100%

Kilograms

Mean =	0.000	
Standard deviation =	0.000	
N =	0	100%

175 Q41C

N =		0	100%
Do not know/Does not apply	8	0	0%
Refused	9	0	0%

176 TQ41A

Please do not fill out or check more than one box. Click on "Next" to revise your answer.

N =		0	100%
	1DI	0	0%

177 TQ41B

Please do not fill out or check more than one box. Click on "Next" to revise your answer.

N =		0	100%
	1DI	0	0%

179 Q44

Which situation best describes your marital status?

N =	0	100%

Married/Common law relationship	01	0	0%
Never married	02	0	0%
Separated	03	0	0%
Divorced	04	0	0%
Widowed	05	0	0%
Other, specify…	96O	0	0%
Refuse	99	0	0%

180 Q45

How many children aged between 0-17 years old do you have living in your household?

Mean =		0.000	
Standard deviation =		0.000	
N =		0	100%
None	8	0	0%
Refuse	9	0	0%

181 Q46A

How long have you lived in your current home residence?

Years

Mean =	0.000	
Standard deviation =	0.000	
N =	0	100%

182 Q46B

Months

Mean =	0.000	
Standard deviation =	0.000	
N =	0	100%

183 Q46BB

Days

Mean =	0.000	
Standard deviation =	0.000	
N =	0	100%

184 Q46B0

N =		0	100%
Refuse	9	0	0%

186 Q46C

About how many hours a day do you spend at home including hours when you

sleep?

Hours		
Mean =	0.000	
Standard deviation =	0.000	
N =	0	100%

187 Q46D

Minutes

Mean =	0.000	
Standard deviation =	0.000	
N =	0	100%

188 Q47

Which of the following groups best describes your ethnic background?

N =		0	100%
White/Caucasian	96	0	0%
Black/African/African-American	2	0	0%
Hispanic/Latino/Spanish	03	0	0%
Asian/Pacific Islander	04	0	0%
Native American/American Indian	05	0	0%
Arab (Middle East, North Africa)	06	0	0%
Indian/Pakistani	07	0	0%
Other	08O	0	0%
I prefer not to answer	09	0	0%

189 Q48

What country were you born in?

N =		0	100%
Canada	01	0	0%
United States	02	0	0%
Other	96O	0	0%
Refuse	99	0	0%

190 Q49

Do you have a driver’s license?

N =		0	100%
Yes	1	0	0%
No	2	0	0%
Do not know/Does not apply	9	0	0%

191 Q50

Do you have access to a motor vehicle in your household (including car, SUV, truck, motorcycle)?

N =		0	100%
Yes	1	0	0%
No	2	0	0%
Do not know/Does not apply	9	0	0%

192 Q51

What is the highest level of education you have completed?

N =		0	100%
Less than elementary school	01	0	0%
Elementary school	02	0	0%
High school	03	0	0%
Community College	04	0	0%
Some university	05	0	0%
Completed University	06	0	0%
Graduate Degree	07	0	0%
Other, please specify	96O	0	0%
Refuse	99	0	0%

193 Q52A

Which of the following best describes your occupational status?

N =		0	100%
Student	01	0	0%
Homemaker	02	0	0%
Unemployed seeking work	03	0	0%
On disability leave	04	0	0%
On parental leave	05	0	0%
Self-employed	06	0	0%
Part-time employed	07	0	0%
Full-time Employed	08	0	0%
Retired	09	0	0%
Other, specify	96O	0	0%
Refuse	99	0	0%

194 Q52B1

What is the name of the neighborhood where you work?

203 Q52C

On an average workday, about how many hours do you spend in your workplace?

Hours	
Mean =	0.000
Standard deviation =	0.000

N =	0	100%

204 Q52D

Minutes

Mean =	0.000	
Standard deviation =	0.000	
N =	0	100%

205 Q53

What is the total yearly income for your entire household before taxes?

N =		0	100%
Under $10000 per year	01	0	0%
Between $10000 and $19999 per year	02	0	0%
Between $20000 and $34999 per year	03	0	0%
Between $35000 and $49999 per year	04	0	0%
Between $50000 and $74999 per year	05	0	0%
Between $75000 and $99999 per year	06	0	0%
Between $100000 and $149999 per year	07	0	0%
Between $150000 and $199999 per year	08	0	0%
Over $200000 per year	09	0	0%
Refuse	99	0	0%

Refuse
